# Male partner involvement in pregnant and breastfeeding women’s use of pre-exposure prophylaxis (PrEP) in a low-income setting in South Africa

**DOI:** 10.21203/rs.3.rs-8590800/v1

**Published:** 2026-01-28

**Authors:** Kamohelo Mangwane, Sarah Schoetz-Dean, Lucia Knight, Victoria Castillo, Lara Court, Kathryn Dovel, Ntobeko Nywagi, Thomas Coates, Landon Myer, Dvora Joseph Davey

**Affiliations:** 1. Division of Social and Behavioural Sciences, School of Public Health, University of Cape Town, Cape Town, South Africa; 2. Department of Epidemiology, Fielding School of Public Health, University of California Los Angeles, Los Angeles, CA, USA; 3. Division of Infectious Diseases, Geffen School of Medicine, University of California Los Angeles, Los Angeles, CA, USA; 4. Division of Epidemiology and Biostatistics, School of Public Health, University of Cape Town, Cape Town, South Africa; 5. School of Public Health, University of the Western Cape, Bellville, South Africa

**Keywords:** Pre-exposure prophylaxis (PrEP), male engagement, pregnancy, postpartum women, HIV prevention, South Africa, qualitative research

## Abstract

HIV in South Africa remains highly gendered, with women experiencing nearly twice the burden of men. Pregnant and breastfeeding women (PBFW) face elevated HIV acquisition risk, and incident maternal HIV infection during these periods increases the likelihood of vertical HIV transmission. Male partners play a critical role in supporting PBFW to initiate and persist on oral pre-exposure prophylaxis (PrEP), yet limited evidence exists on how best to foster partner support.

We conducted in-depth interviews with male partners of PBFW enrolled in a randomized trial (SCOPE-PP) that offered real-time biofeedback counselling on PrEP adherence and access to HIV self-testing (HIVST) in both study arms. Eligible male partners of participants in the intervention arm were invited to participate in November-December 2024. A thematic approach explored men’s perceptions of and support for their partners’ PrEP use.

We completed sixteen interviews. Median relationship length was six years; four partners were married to the index participant. All PBFW were taking PrEP at the time of the interview. Men generally held positive views of PrEP, describing it as protective for both mother and infant. Many provided emotional encouragement or practical support. Open communication helped resolve initial mistrust or gendered assumptions that women’s PrEP use signaled suspicion of male infidelity. Exposure to PrEP also prompted men to reflect on their own HIV prevention needs.

Findings underscore the value of approaches that center communication within couples to strengthen disclosure and male engagement in PrEP uptake and adherence during the perinatal period and to promote shared responsibility for HIV prevention within families.

## INTRODUCTION

South Africa has historically carried the largest burden of the global HIV epidemic, with nearly 1 in 8 individuals within the general population living with HIV [1]. Efforts made over the past decade, including the expansion of testing and treatment coverage, have led to a significant reduction in AIDS-related mortality [2]. Despite progress, the spread of HIV remains uncontained due to gaps in HIV prevention strategies among key demographic groups [3]. The impact of HIV within South Africa is skewed considerably by gender, with prevalence in women nearly twice that of their male peers [4,5]. The risk of HIV acquisition among pregnant and breastfeeding women is nearly double that of non-pregnant or postpartum women [6,7] and women with acute HIV infection during pregnancy or breastfeeding periods have an elevated risk of vertical HIV transmission [8].

Recognizing this increased risk, the World Health Organization explicitly recommends that pregnant and breastfeeding women (PBFW) are provided with oral pre-exposure prophylaxis (PrEP), and most recently long-acting injectable PREP, which has demonstrated high effectiveness in reducing the risk of HIV acquisition [9] [10]. However, the uptake and continued use of PrEP among South African PBFW is suboptimal, often impeded by a unique blend of socio-ecological factors which have been well-illustrated in prior research, including those related to support and sexual relationships with their partners. A recent study revealed that women who were married or cohabiting with their partners or reported intimate partner violence were less likely to remain adherent to daily oral PrEP [11]. Women linked their partners’ behaviour to their perceptions of whether their risk of HIV acquisition was heightened or reduced and highlighted the importance of partner testing, education and counselling alongside their PrEP use [12]. Additionally, male partners living with HIV in serodiscordant relationships, also described their partners’ oral PrEP use as beneficial, as it supported their own adherence to antiretroviral treatment (ART) [13]. These partners showed their support for their partner’s PrEP use by providing reminders and encouragement. Social support and male partner involvement in other HIV-related care in Sub-Saharan Africa (SSA), including vertical HIV transmission prevention (VTP) and Early Infant Diagnosis (EID), have been associated with positive health outcomes [14]. Another study in Malawi demonstrated that male partners were receptive to the use of daily oral PrEP by their partners because of the protection it offered from HIV acquisition for themselves and their infants [13].

Despite this and recent research on male partners of women generally, few studies examine the attitudes and beliefs about PrEP among heterosexual partners of PBFW. This study explores the perspective and experiences of male partners who provide social support to PBFW using oral PrEP. The data were collected as part of the *Stepped Care to Optimize PrEP Effectiveness in Pregnant and Postpartum women* (SCOPE-PP) parent trial. Findings contribute evidence on relationship dynamics and men’s perceptions within the context of PBFW’s PrEP use, with implications for improving HIV prevention strategies during pregnancy and breastfeeding.

## METHODS

### Study design

The qualitative exploratory study reported here is nested within the SCOPE-PP parent study (R01HD106862; NCT#05322629), described in greater detail previously [16]. Briefly, the study aimed to understand the impact of the SCOPE-PP interventions, including real-time biofeedback counselling on recent PrEP use and the offer of an HIV self-test (HIVST), on PrEP adherence among pregnant and postpartum women living without HIV. Researchers screened a subset of SCOPE-PP participants enrolled in the intervention arm to assess the eligibility of male partners. Male partner eligibility criteria included partners of PBFW in the SCOPE-PP study who were comfortable and available to attend the interview in the study clinical site in Cape Town. Female participants with eligible male partners provided written consent. Once written consent was obtained, a physical flyer (Figure I) [17] was shared to facilitate partner recruitment for the qualitative sub-study. Ethical approval for SCOPE-PP was obtained from the University of California, Los Angeles Institutional Review Board (IRB# 21-1770) and the University of Cape Town Human Research Ethics Committee (HREC117/2022) prior to study commencement. Male participants received a R100 (~ $5 USD) voucher alongside a R50 (~$2.50 USD) transportation voucher for their time and transportation to the study site.

#### Data collection and analysis

Due to the sensitive and personal nature of the interviews, a male South African researcher, trained in qualitative methods, was selected to conduct the interviews (NN). The interviews were held at a local health facility, except for one held in an office at the University of Cape Town and were approximately 40 minutes long. Audio-recorded interviews were conducted in participants’ first language, isiXhosa. Topics covered in interviews included: (1) relationship dynamics, (2) perceptions of maternal PrEP use (including acceptability and support) and (3) male partners’ experiences and opinions regarding HIV prevention and treatment services, including HIVST understanding and use. Audio recordings were then transcribed verbatim and translated into English for analysis. To guarantee participant privacy and confidentiality, all interviews were de-identified for transcription and storage.

A thematic approach was applied to interpret results, including a hybrid of inductive and deductive reasoning for initial codebook development, with the in-depth interview guide serving as a grounding template [18]. Following initial familiarisation with the transcripts, a team of three South African and American female researchers (KM, SSD, VC) coded and analysed transcripts using qualitative software (Dedoose Version 9.0.86; www.dedoose.com). To safeguard the trustworthiness and rigour of the work, researchers strictly followed the four criteria commonly applied in qualitative research: credibility, dependability, confirmability, and transferability [19]. To establish intercoder reliability as well as iteratively refine the codebook, the initial transcripts (n=3) were reviewed in unison using a parallel double-coding approach. After establishing codebook comprehensiveness and addressing necessary reconciliation, the remaining transcripts (n=13) were divided and independently coded by the same three researchers. Researchers kept an ongoing audit trail and held weekly meetings to facilitate reflexivity and discuss emergent themes throughout analysis [20,21] .

Grounded in health promotion and social support theory, our analysis conceptualizes social support as the functional and qualitative aspects of the relationship between male partners and PBFW [15], enabling us to examine how male partner involvement influences maternal PrEP uptake and adherence, while also recognizing the broader social and structural factors that shape women’s access to and continued use of PrEP in this context. Our analysis of male partner experiences with PBFW’s PrEP use is described using an adapted version of the Social Support Framework [22] (Figure II). The original framework highlights the importance of support from one’s social network in maintaining an individual’s health and well-being.

It describes four different forms of support: (1) Tangible (instrumental) support is defined as practical, concrete and direct material assistance (e.g., providing transport or financial assistance) [22]. (2) Emotional support focuses on empathy and providing an increased sense of acceptance and self-worth (e.g., providing reassurance, comfort, listening and offering emotional safety [15,23]). (3) Informational support is discussed as the provision of knowledge about PrEP use in the form of feedback, guidance or advice, while (4) Social companionship is defined as time spent together in leisure or recreational activities [24]. While the framework describes these forms of support in well-defined categories, true examples often overlap into more than one category. The original framework was adapted for use in analysis to include contextual components that men considered important. These components interacted with and influenced the social support men offered to PBFW. The broader social context, specifically social networks (defined as networks of social relationships with people [25], including the male partner, family and children) was included. The role of the relational context was also considered, as men felt that trust *in* and *from* their partner was important, as well as their living arrangements.

## RESULTS

A total of 46 recruitment flyers were distributed by PBFW participants in the study to recruit male partners, from which we were able to contact, screen and interview 16 men in one-on-one semi-structured in-depth interviews between 18 November 2024-12 December 2024. All participants provided written informed consent. Men mostly lived with their female partners (12/16) and had long-term relationships (>1-2 years) (14/16). All female partners were postpartum (16/16) and actively in the parent trial at the time of interviews. A total of 14/16 men reported not living with HIV at the time of the interview, of whom one was taking PrEP; two men reported to be living with HIV and were taking ART.

### i Men’s Perception and Experience with Partners’ PrEP Use

Participants described firsthand experiences of their partners’ PrEP use along a spectrum, ranging from negative to positive. Men in the sample largely reached positive conclusions about their partner’s PrEP use over time. As with their reasons for support, described below, this was often linked to the protection PrEP provided. Men viewed their partners’ decision to take PrEP as an act of service for the family. This led men to view their partners positively, praising and appreciating them for their sense of responsibility, as noted by one man who highlighted the positive example his partner set for their children.

*We, as parents, are role models. Everything that we do, they can see and copy it from the best. So, everything that we do and something that we advise one another about, we even go further advising children, so, that’s why I mention this part about children…Something that I like is her valuing of time and the discipline at home, yes, and the order*. (Unknown HIV status in a 7-year relationship, living together for 6 years)

Other men described initial hesitance and concerns relating to questions of trust and infidelity. Some men felt this decision was related to the female partner’s lack of trust in them, while others interpreted it as an indicator that their female partner had other sex partners. This participant explained how he felt that he was untrusted due to his partner’s PrEP use.

*To be honest, I didn’t feel good when I saw her using the pill, it felt like she didn’t trust me and that I was the one who was sick, you see. It felt like it was me, because I was the one who was cheating*. (HIV-negative in a 7-year relationship, married for 4 years)

This perception that they caused their female partner to perceive herself as facing increased risk of HIV, spurring her decision to take PrEP, resulted in a negative reaction from the male partner. This was echoed by another participant, who, although acknowledging the benefits of PrEP for his female partner, initially felt challenged by his partner’s PrEP use. This facilitated an in-depth conversation about their sexual health.

*Yes, from the beginning, it was not easy because I did not know what her objectives were, but I knew this process was heading towards protection. There was this thing that she needed to know, or we had to look after our health. After knowing and [I] ended up learning more about PrEP, at least I became free*. (HIV-negative in a 3-year relationship, living together for 2 years)

Extensive and in-depth discussions about PrEP use while pregnant or postpartum, while uncomfortable for some, often eased the initial hesitance men had and created opportunities to probe their partner’s motivations for initiating PrEP. These conversations resulted in both greater support and recognition of the value of PrEP, as well as potentially stimulated important conversations about relationships between couples, strengthening these. Conversations also facilitated access to information about HIV prevention and greater awareness about PrEP and its benefits, as described by one man who was able to have a greater understanding and acceptance of his partner’s PrEP use.

*I did not have much of a problem, because another way that a person can take this is that it can be a problem, that it may look like you are not trusted. That is, why is she taking this PrEP, because that may cause [an] argument. When I opened my mind, I did think that there is a lot that is happening outside, because it can also protect me, because there are things that are happening like rape outside. If that happens, I will also be protected... If she can protect herself, it means that she also protects me*. (HIV-negative in a 12-year relationship, living together for 10 years)

In one case, this discussion confirmed one man’s suspicion that him having other sex partners influenced his partner’s decision, but he also seemed to feel that she had made a good choice and was happy that she was protected.

*She told me, she brought the pills when she was pregnant and got them here. She said they were for PrEP. I didn’t understand that. I asked her to tell me exactly what they were. She said okay, these pills will protect her, since I knew I was a cheater. So, if I had sex with someone who has HIV, they will protect her and me from getting infected*. (HIV-negative in a 7-year relationship, married for 4 years)

Men described how these conversations resulting from their female partner starting PrEP not only resolved initial negative reactions, but also strengthened their relationships and men’s support of their partners.

*I was shocked. I wondered if she didn’t trust me or if she saw something wrong with me.… We talked about it, I listened to the way she explained it to me, then I was satisfied*. (HIV-negative in a 7-year relationship, married for 4 years)

Another participant explained how the discussion fostered unity with his partner and shifted his mindset.

*We usually have discussions that I think they are…disciplining me…because something that I notice is that she made me to be on the same mind with her*. (HIV-negative in a 6-year relationship, living together for 6 years)

Men felt that trust in their partner as well as from their partner was an important issue. They similarly thought that their living arrangements were important. These contextual components interacted with and influenced the social support men offered to PBFW. Male partners’ ideas on how support should be offered to PBFW using PrEP, how they offered support to their own partners and their reasons for their chosen forms of support are discussed. These ideas often reflected how they provided support to their partners as they referenced their own support in discussions. Additionally, the impact of their partners’ PrEP use on trust in their relationship and male partners’ thoughts about their own engagement with health-related activities are explored.

### ii Provision of support

Male partners provided various forms of tangible and emotional social support, often overlapping with companionship, to help PBFW remain engaged with health services and adhere to PrEP. One man described providing overlapping forms of tangible (i.e., reminders and financial support for transport), as well as emotional support and companionship (i.e., by accompanying his partner to the clinic).

*She did not have a cellphone for a long time so they would call from my phone and then I would give a message telling her that they phoned there from the clinic…when she does not have money to come here at the clinic I’m able to find something and give her money…Or if I do not have money then I’m able to escort her and come here together*. (Unknown HIV status in a 7-year relationship, living with partner for 6 years)

Several men noted that attending clinic visits required a temporary lapse in what they saw as women’s household duties or time spent with partners and families. One partner, who took over childcare during clinic visits, explained that this was a form of tangible support:

*The one challenge is that I am the parent who is available at home. For example, when she is stepping out, I must be available for the children at home. Since the children are still young, we give each other a chance*. (HIV-negative in a 12-year relationship, living with partner for 7 years)

Another participant noted his availability as important in facilitating his partner’s access and assisting her to overcome potential barriers to PrEP use, such as a loss of time for work and other household responsibilities. He then explained that he also provided emotional support in the form of encouragement, checking in with her and offering reminders to attend her clinic appointments.

*All I do is check on her since I’m busy with work. So, I encourage her to just give her the pills. I check on her when she goes to the clinic. And when she comes back, I check on her…I make sure that she has gone to the clinic*. (HIV-negative in a 6-year relationship, living with partner for 3 years)

When exploring the reasons that male partners gave for providing support, many acknowledged the importance of their partners’ PrEP use. Many men described PrEP as valuable, as it offered reliable protection from HIV for the woman, themselves and the rest of their family.

One participant explained that his confidence in PrEP stemmed from community-wide awareness of its efficacy for prevention, motivating him to support his partner’s continued use:

*What made me support my partner in using PrEP, bro, I really like it I know how PrEP works because it works very well and protects against HIV. People have also talked about it, they praise how PrEP works. That’s what makes me happy, especially when I see my partner, who I live with, using PrEP, and people are talking about it positively*. (HIV-negative in a 2-year relationship, living together for 2 years)

This focus on protection resulted in PrEP use being positively framed by the participant. Some men specifically noted that PrEP offered protection if they, or their female partner, had other sex partners or sexually assaulted, and how this motivated encouragement of their partner’s PrEP use. Another participant noted how his support was motivated by knowledge that his partner was taking steps to protect herself from HIV in their serodiscordant relationship.

*It is because of the way I fell sick [with HIV], and that is why I am supporting her. I do not wish her to be the same as I was*. (Living with HIV and married, living together for 5 years)

Another participant explained how his support of his partner’s PrEP use was driven by perceptions that her PrEP use compensated for his lack of consistent care or consideration of his own health, including HIV testing.

*The thing that makes me support my partner is because I am not around too much about these things, like checking health, like, every time after 3 months, checking my status, you see*. (HIV-negative in a 3-year relationship, living together for 2 years)

Furthermore, men discussed fearing being perceived as being unsupportive, especially by health care workers (HCWs), motivating them to support their partners in keeping a consistent routine of care. Men who offered non-resistance as support thus did so not only because they viewed it as a viable form of support, but because it offered a passive way to avoid being seen as unsupportive by HCWs.

*I just thought that if maybe it can happen, she misses her date, people might say ‘Hey, it is because of that [man] you were with the other day, maybe he does not want her to come to the clinic’…Whereas the clinic is the place where people get help. I also drive her and say, ‘My friend, this is the time for you to go to the clinic. You must not miss your date’*. (Living with HIV, married, living together for 5 years)

Men also described how their partner’s involvement in the study and being on PrEP offered a low-effort way to access health information, as their partners brought home information gathered during clinic visits. Here, one man who had recently been diagnosed with HIV explained how his partner’s engagement was an avenue for him to learn more about his own HIV.

*Something that I like is the place where she goes. If she finds out that I am also supposed to go there, she takes me so that I can come as well in the same place. So that I can get more information because I don’t have much knowledge. It is not for a long time that I have HIV*. (Living with HIV, married, living together for 5 years)

Men consistently recognized that the use of PrEP was both worthy and significant, with advantages that extended beyond their partners, which created incentives to provide support. These perceptions influenced how men understood their relationships, including trust and support, and shaped their own approaches to health engagement.

### iii Impact of PBFW PrEP Use on Men’s Engagement with Their Health

Men in the study acknowledged that they were often poorly engaged with their own health. They believed that it is difficult for men to integrate PrEP use and health-related activities into their daily lives, as emphasized by one man who explained men’s failure to consistently recognize the health implications of their actions.

*‘I can’t wake up in the morning for the clinic’. That’s a guy’s mindset. Let’s say I could sleep with a girl without using a condom, but I’m suspicious of her, but I’m not going to wake up and run to the clinic*. (HIV-negative in a 2-year relationship, living together 2 months)

He also highlighted the lack of priority that men afford to attending health services. Despite the challenges he noted about male engagement in health care, he went on to make a point about the merit of PrEP in situations of infidelity, where men might otherwise experience lingering uncertainty.

*But if you know the person you had sex with is HIV-positive, if you use PrEP, you can have sex with this person, but not intentionally, you have sex with her, and you do not know each other. When you hear on the following day that [the] person has HIV. You will have your doubts but knowing that “I have used PrEP”*. (HIV-negative in a 2-year relationship, newly living together under 2 months)

This idea of PrEP’s value in scenarios of infidelity or non-monogamy was echoed by other men. Despite this recognition, many men also provided reasons that they would not use PrEP themselves.

*[For] other men who know themselves and their ways, [PrEP] can be alright for them. Yes, also to decrease the numbers of HIV infection, this can be alright. For people who know that they have other things that they are doing [sex outside the relationship], but for a man like me, I know that there is nothing else I am going to do. So, there is no problem*. (HIV-negative in a 2-year relationship, living together for 2 months)

Other reasons that men expressed they would not be able to use PrEP were that they felt that they would face some of the same challenges accessing facilities as women, such as home and work responsibilities, highlighted in the sections discussing men’s support above. They also highlighted day-to-day reasons, such as pill burden, which impacted their decision not to take PrEP.

*I would like to use PrEP, but the problem is that I do not like to use a lot of pills. Then it would be better for me if I were to receive it as an injectable, maybe once in a month, then I can be able to use PrEP*. (HIV-negative in a 3-year relationship, living together for 2 years)

Despite providing reasons not to use it, most men did say they would consider using PrEP, with interest sometimes stimulated by their partner’s use.

*I ended up seeing the reasons for me coming here because I could see that there is help from using PrEP. Because there is prevention from something that you do not know when you could get it…Then I saw the way she was doing it whenever she would come back from this study and how she was taking me through, and then I felt like it could be okay. There could be something that I can benefit from as well if I can go and listen about this thing of PrEP*. (HIV-negative in a 2-year relationship, newly living together under 2 months)

Despite this, only one of the men had ever used PrEP, and therefore most had never actively sought it out. Despite their admiration of their partners’ sense of responsibility in relation to their use of PrEP, male partners largely did not consider shouldering any of this same responsibility. While acknowledging men’s overall lack of healthcare engagement, limited participation was often connected to the concept of trust and the belief that men who used PrEP or frequently tested for HIV did so because they had other sex partners. Inversely, several participants correlated their partners’ PrEP use to a general lack of trust in men.

## DISCUSSION

This study explores male partners’ perspectives and experiences with daily oral PrEP use by their pregnant and postpartum partners. The introduction of PrEP yielded perceptions that men themselves were not trusted. This extended to gendered perceptions around the trustworthiness of men and beliefs that this was the reason women were initiated on PrEP. Previous findings in Southern Africa showed that men had a heightened perception of HIV risk, while women attributed their risk to their male partners’ sexual behaviours [30, 31, 37]. Nonetheless, the presence or absence of trust for both men and women played a key role in guiding decisions about their health behaviours, including motivation to use PrEP [31, 37]. Recent studies have shown that disclosure of PrEP to partners increased social support and was associated with improved PrEP adherence [30, 31, 33,34]. In our study, extended discussions often eased initial mistrust and resulted in acceptance and support from men and sometimes strengthened relationships. In some cases, particularly for men with other sex partners, these discussions confirmed suspicions that their sexual activity influenced their partners’ decision to use PrEP.

Anticipated stigma from men’s social networks has previously been identified as a barrier to PrEP use among women [26]. Although anticipated stigma was not directly addressed by male participants in our study, men feared being seen as unsupportive by HCWs and offered support to prevent this perception. Men provided examples of the tangible and emotional support, and companionship, they offered to assist their female partners to engage with PrEP. Male partners supported their partners emotionally and tangibly by providing reminders and offering encouragement [11, 31]. Tangible support was provided to overcome barriers that their partners faced in accessing health facilities that could potentially impact their adherence. Previous findings found similar barriers related to communication with and access to facilities, including competing priorities or responsibilities that may limit women’s access to services and adherence to PrEP [26–30]. In response to these barriers, men described providing transport or financial support, as well as in-kind support such as providing childcare, taking messages or accompanying their partner to the facility.

Male partners had a range of reasons for providing support for their partners, the most common being that they valued the protection from HIV that PrEP offered their female partners, themselves and their infants/children. Taking up this responsibility to protect the family also fed into men’s general positive perception of their partners’ PrEP use. Most men in the study said that they would theoretically be willing to use PrEP; however, they provided reasons why PrEP use was not suitable for their lifestyle. Given this, male partners were incentivised not to resist their partners’ PrEP use and to provide support. Likewise, Zimbabwean male partners had positive attitudes towards PrEP use when they thought it would benefit them in casual relationships, but were less supportive in long-term relationships, where they thought PrEP use threatened their ability to control their partners’ sexuality [32]. Like our study, where male partners were or had the intent for long-term relationships, this highlights the intent to shift the responsibility for joint health issues to women by male partners in relationships. Unlike the Zimbabwean study, this led to partner support and increased positive perception of PBFW partners due to their taking over this responsibility in long-term relationships in our study.

PBFW’s PrEP use also prompted their male partners to consider their own health engagement and behaviour. They often preferred supporting their partners’ PrEP use, despite reported willingness to use PrEP, as they valued its benefits but felt that it was not suitable for their lifestyles. In general, they believed that men were poorly engaged in health matters, and discussions about PrEP use prompted them to interrogate their own health engagement and sexual behaviour. Poor male engagement in HIV care has been associated with societal gender norms, persistent HIV stigma and the competing priorities of employment in South Africa [35]. Insights into their willingness to initiate PrEP themselves were consistent with findings that have shown that men’s risk perception and thus motivation to use PrEP is often linked to mistrust in their relationship [36].

The limitations of this study include limited transferability due to the application of broad eligibility criteria, which likely led to a homogenous set of views, including views only from men whose partners invited them into the study. Male participants who were enrolled to participate in the interview were those who were interested in the study. Additionally, due the nature of the recruitment, their partners would have likely disclosed their PrEP use, leading to positive views in support of PrEP use by their partners being potentially overrepresented.

## CONCLUSION

Male partner support of PrEP use among their pregnant and postpartum partners is influenced by gender ideals, desires to establish trust in the relationship and a preference for women to take over the responsibility of protecting the family from contracting HIV. In-depth discussions about PrEP use play a key role in addressing general perceptions that men are untrusted and encourage men to consider their own health behaviour and risk perception for contracting HIV. Individual trust in romantic relationships also influences the provision of support by male partners, who feared being seen as unsupportive and disengaged by health care workers. Limited male engagement in health matters is a key concern identified by male partners who mostly agree on the need for male involvement in their partners’ PrEP use during pregnancy and postpartum periods. These findings highlight the importance of disclosure of PrEP use combined with open communication, shared responsibility, and greater male engagement in HIV prevention during the perinatal period and to support PrEP adherence within families.

## Figures and Tables

**Figure I:**
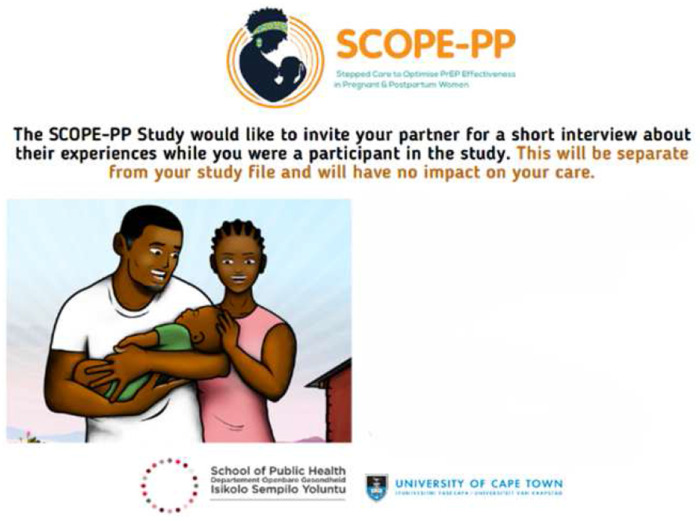
Male Partner In-depth Interview Invitations provided to SCOPE-PP participants (Adapted from Mphande et al., 2025 [17]).

**Figure III:**
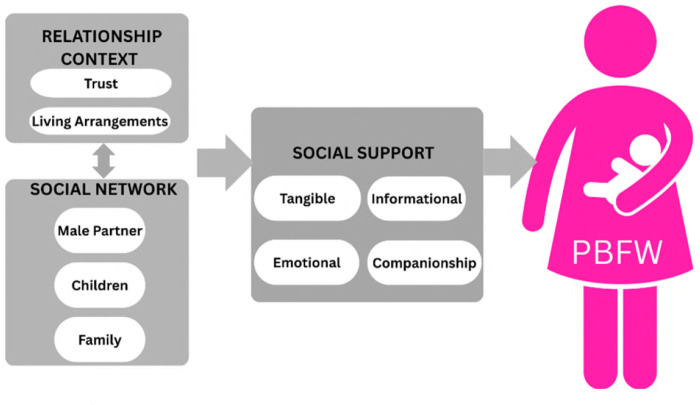
Schematic of Social Support among Pregnant and Breastfeeding Women using PrEP and Receiving Adherence Biofeedback PrEP Counseling (SCOPE-PP Intervention)

## Data Availability

The dataset supporting the conclusions of this article is available upon request from the study PI, Dr. Dvora Joseph Davey at dvoradavey@ucla.edu.
